# Precisely Printing Perovskite Nanocrystals in Glass via Thermoelectric Synergistic Effect

**DOI:** 10.1002/advs.75827

**Published:** 2026-05-26

**Authors:** Zhiheng Miao, Yao Zhou, Minzhi Li, Zhe Wang, Xiaolong Yu, Jianlin Li, Changjiu Li

**Affiliations:** ^1^ State Key Laboratory of Tropic Ocean Engineering Materials and Materials Evaluation Hainan University Haikou China; ^2^ College of Materials Science and Engineering Hainan University Haikou China; ^3^ School of Ecology Hainan University Haikou China

**Keywords:** CsPbX_3_ nanocrystals, glass, printing, thermoelectric treatment

## Abstract

CsPbX_3_ (X═Cl, Br, and I) perovskite nanocrystals (PNCs) embedded glass exhibit excellent stability and optical properties, yet the patterned distribution of CsPbX_3_ PNCs in glass remains challenge. In this study, precise distribution control of CsPbBr_3_ PNCs in glass was successfully achieved through thermoelectric synergistic treatment. Upon thermal‐electric fields, the structure and elemental distribution in the affected region of the glass undergo rearrangement. The migration and enrichment of Cs^+^ in the glass are the primary reasons for precipitation of CsPbBr_3_ PNCs and dependent on temperature, electric field strength, and duration. By regulating the temperature, electric field strength, and duration during thermoelectric processing, the depth and thickness of the luminescent layer of CsPbBr_3_ PNCs can be precisely controlled at the micrometer scale. Furthermore, by utilizing patterned electrodes, we achieved a consistent patterned distribution of CsPbBr_3_ PNCs in glass, as well as precise control at the micrometer scale. This method opens up a new avenue for precise printing and crystallization regulation of CsPbX_3_ PNCs in glass.

## Graphical abstract

Based on the direct migration and enrichment of Cs ions in glass driven by thermoelectric fields, a novel method has been developed to achieve controllable crystallization and patterned distribution of CsPbBr_3_ PNCs in stable glass. The distribution scale of PNCs strongly depends on the thermoelectric processing conditions (temperature, electric field, and duration).

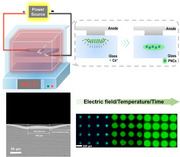

## 1 Introduction

CsPbX_3_ (X = Cl, Br and I) perovskite nanocrystals (PNCs) have emerged as candidate materials for next‐generation optoelectronic devices, owing to their tunable bandgap, near‐100% quantum yield, and excellent photoelectric conversion efficiency [1, 2, 3, 4]. However, these materials are inherently sensitive to water, oxygen, and heat, severely limiting their practical applications [5, 6, 7]. To address this issue, an excellent solution has been proposed: embedding CsPbX_3_ PNCs into an inorganic glass matrix [8, 9]. The dense network structure of the glass can effectively isolate PNCs from external erosion, while retaining the inherent photoelectric properties of the CsPbX_3_ PNCs [10, 11, 12]. The photoluminescence quantum yields (PL QYs) of CsPbX_3_ PNCs in glass have surpassed 90% [10, 11, 12]. After enduring a harsh aging test at 85°C/85% RH for 50 days, the photo‐luminescence intensity of CsPbX_3_ PNCs embedded in glass still remains above 90% [12]. By designing PNCs embedded glass as a backlight source, the LCD device exhibited a wide color gamut, covering 127% of the NTSC standard and 176% of commercial LCDs [12].

Currently, achieving a controllable patterned distribution of PNCs represents the core challenge for the functionalization of this glass composite [13, 14]. The spatial distribution pattern of PNCs directly determines the application and performance of the device such as Micro‐LED, information storage, and biological imaging [15, 16, 17]. However, the current mainstream regulation methods exhibit significant limitations. Traditional thermal treatment methods facilitate the crystallization of perovskite precursors in glass through overall heating [10, 11, 12]. While this approach is simple to implement, it is constrained by the isotropy of heat conduction. Nanocrystals tend to form irregular agglomerates inside the glass, making it challenging to achieve regional distribution regulation. Therefore, developing a crystallization regulation technology that combines high precision and practicality has become the key to promoting the industrialization of CsPbX_3_ PNCs embedded glass composite.

The migration and local aggregation of elements Cs, Pb, and X are of significant importance in the precipitation and spatial distribution of CsPbX_3_ perovskite nanocrystals (PNCs) [18, 19, 20, 21, 22]. Considering that a single thermal field is unable to regulate the migration of Cs, Pb, and X, the introduction of external fields may represent a potential strategy for regulating the crystallization behavior and spatial distribution of CsPbX_3_ PNCs. During material processing, the introduction of an external electric field can induce the directional migration of ions within the material, accompanied by structural reconstruction, thereby regulating material properties such as mechanical properties, adhesion, and dielectric properties [23, 24, 25]. The use of structured or embossed electrodes can result in a non—uniform three—dimensional distribution of the electric field, leading to non—uniform patterning modulation of the material's structure and properties. With the advancement of micro—nano processing technology, precise processing and modification of materials at the micro—nano scale can be achieved by controlling parameters such as electrode shape and applied voltage [26, 27]. Coupled with the low cost of this processing method, it offers broad prospects in material functionalization.

Consequently, this study proposes a strategy based on thermoelectric synergistic effects to induce the precipitation and spatial distribution control of CsPbX_3_ PNCs in glass. The thermoelectric field was utilized to achieve the migration and enrichment of Cs^+^, Na^+^, Br^−^, and O^2−^ elements in glass, thereby successfully inducing the precipitation of CsPbBr_3_ PNCs in the glass. By regulating the intensity of the applied electric field, heating temperature, and processing time, precisely control of the distribution depth, distribution area, and optical properties of CsPbBr_3_ PNCs in the glass can be achieved. The localized electric field generated by structured electrodes enables effective regulation of the spatial distribution of CsPbBr_3_ PNCs in the glass, thus allowing for micron‐scale luminescent patterns. This method offers a novel approach for the low‐cost, high‐precision precipitation and distribution control of CsPbBr_3_ PNCs in glass, holding promise for advancing the practical application of CsPbX_3_‐based optoelectronic devices in areas like High‐definition display and information storage.

## 2 Results and Discussion

**FIGURE 1:**
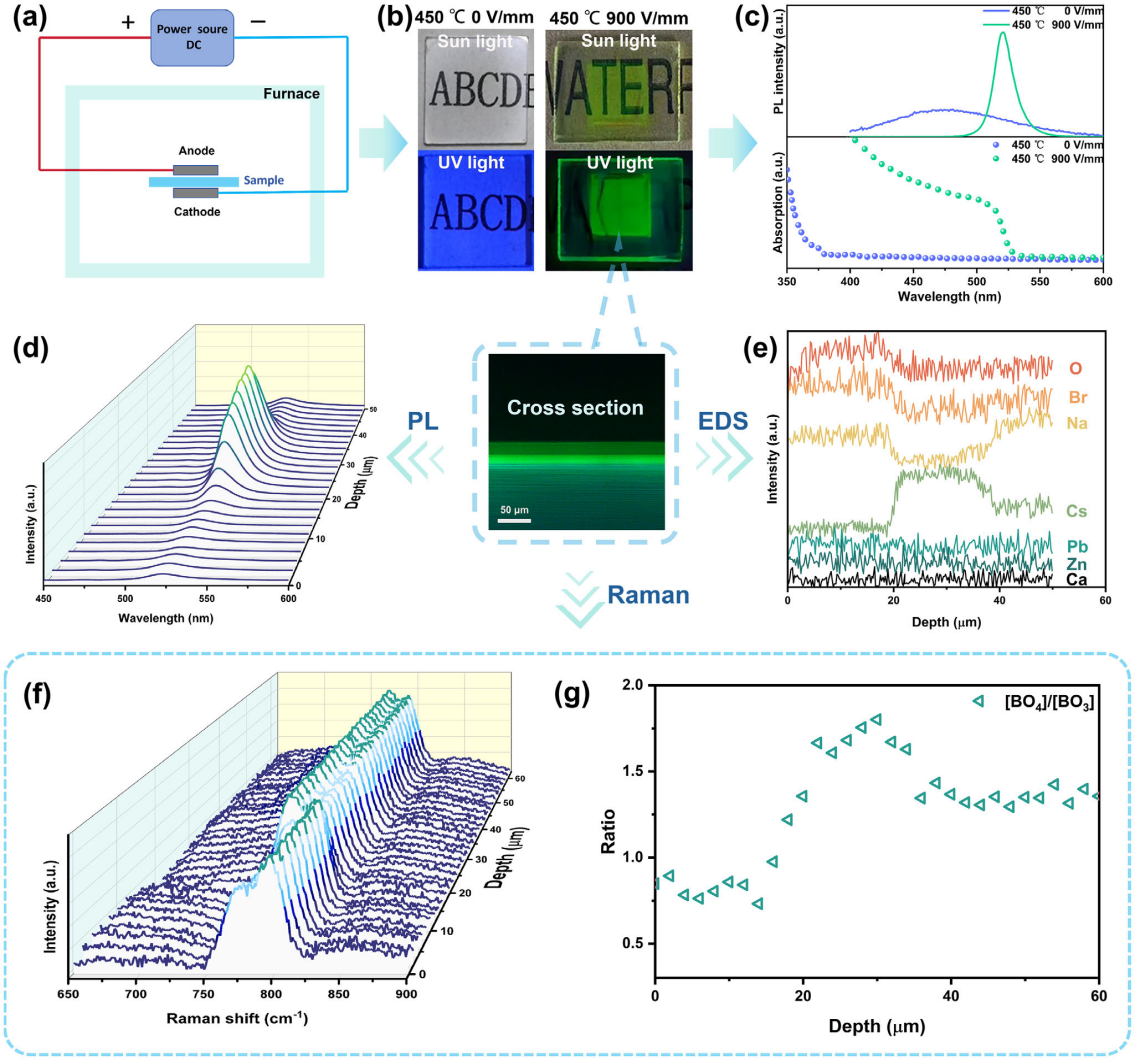
(a) Schematic diagram of the thermoelectric treatment device. (b) Photos of glass samples after heat treatment and thermoelectric coupling treatment, taken under sunlight and 365 nm ultraviolet light. The photo within the dashed box depicts a cross‐sectional image of the glass area that underwent thermoelectric treatment. (c) Emission and absorption spectra of the glass samples after heat treatment and thermoelectric coupling treatment. (d) Micro‐area photoluminescence spectra, (e) Distribution of elements, (f) Raman spectra of glass area subjected to thermoelectric treatment are collected at different depths spanning from 0 to 60 µm. (g) The ratio of the absorption peak areas corresponding to the vibrations of [BO_4_] and [BO_3_] with depth.

In this study, a glass composition capable of precipitating CsPbBr_3_ PNCs was chosen (XRD results shown in Figure S1). The effect of thermoelectric treatment on the precipitation and distribution of CsPbBr_3_ crystals on the anode side was investigated at the temperatures below the crystallization temperature of CsPbBr_3_ in glass (Figure S2). For samples subjected solely to thermal treatment, no characteristic absorption or photoluminescence (PL) peaks specific to CsPbBr_3_ PNCs were observed at temperatures below 450°C. The samples treated at 460°C displayed only exceedingly faint characteristic PL peaks along with absorption shoulder peaks. The device used for glass thermoelectric treatment is depicted in Figure 1a and Figure S3. The glass sample without thermoelectric field treatment (450°C, 0 V/mm) appears colorless and exhibits good transparency. The blue luminescence observed under ultraviolet (UV) irradiation arises from the 6s6p‐6s^2^ transition emission of Pb^2+^ ions in the glass [28, 29], as demonstrated in Figure 1b. The glass sample treated with a thermoelectric field (450°C, 900 V/mm) exhibits a light green square area (very close to the shape and size of the electrode plate) under sunlight. The square area of this sample emits bright green light under UV irradiation. The absorption and photoluminescence (PL) spectra of the sample treated with and without a thermoelectric field are presented in Figure 1c. The glass sample heat‐treated solely at 450°C exhibits no absorption peak in the visible light range from 380 to 600 nm, and its broad PL peak at 475 nm attributed to Pb^2+^ ions in the glass matrix. For the sample post‐thermoelectric treatment, absorption shoulder peak and narrow‐band PL peak located at ∼520 nm is observable, aligning with the bandgap of CsPbBr_3_ PNCs [30, 31], indicating their precipitation in the electric field effect region of the glass. The thermoelectric treatment temperature (450°C) is lower than the crystallization temperature of CsPbBr_3_ PNCs under simple heat treatment conditions (470°C, Figure S2). The cross‐sectional view of the thermoelectric field effect region in Figure 1b reveals that the luminescent region is located at a depth range of ∼20‐40 µm below the glass surface. The PL spectra at different depths also indicate that the photoluminescence region is within a depth range of ∼20‐40 µm (Figure 1d). The precipitation of CsPbBr_3_ PNCs in the thermoelectric field effect region of the glass is closely correlated with ion migration in the glass. A continuous rise in current within the circuit suggests the migration of ions inside the glass (see Figure S4). Figure 1e illustrates the distribution of elements with depth in the thermoelectric effect region on the positive side of the glass. Notably, the Cs element is enriched at a depth of 20–40 µm, aligning with the depth range of the luminescent region, suggesting that the precipitation of CsPbBr_3_ PNCs in this region is triggered by the enrichment of Cs element. The enrichment of Cs is attributed to the migration of Cs ions under the influence of an electric field. With the electric field, Cs ions on the glass surface migrate inward, leading to a depletion of Cs ions on the surface and an elevation of concentration in the inner layers of the glass. This observation was further corroborated in the backscattering image of the glass cross‐section (Figure S5), where the enrichment of Cs with a large atomic number resulted in striped bright areas inside the glass, while the depletion of Cs caused the surface area of the glass to darken. Conversely to the migration direction of Cs ions, Br^−^ and O^2−^ anions accumulate on the glass surface under the electric field. Due to its higher electronegativity, Pb^2+^ has a higher diffusion barrier, making it difficult to migrate under an electric field. Consequently, its concentration remains consistent with depth (see Figure 1e). It is worth noting that after exposure to a thermoelectric field, Na ions are relatively scarce in the areas where Cs ions are enriched, while the concentration of Na ions on the surface of the glass increases. This is because the migration of Cs cations and Br^−^ and O^2−^ anions creates a charge imbalance between the surface and the interior of the glass [32, 33]. Since Na^+^ has a smaller ionic radius and a lower diffusion barrier, it has good mobility and is more easily migrated to the surface of the glass, playing a role in balancing the charges.

To further investigate the structural changes in the glass network caused by ion migration under thermoelectric effects, we tested the micro‐area Raman spectra at different depths of the glass surface, as shown in Figure 1f. The absorption peak at 775 cm^−1^ originates from the stretching vibration of [BO_4_], while the absorption peak at 810 cm^−1^ comes from the stretching vibration of [BO_3_] [30, 34]. As the depth increases, the intensities of the absorption peaks at 775 and 810 cm^−1^ undergo significant changes (see Figure 1f and Figure S6), suggesting that ion migration under an electric field induces structural changes in the glass network. Figure 1g depicts the variation of the intensity ratio of the absorption peaks at 775 and 810 cm^−1^ with depth. The intensity ratio of the absorption peaks remains essentially unchanged at depths ranging from 0 to 20 µm below the glass surface, but significantly decreases at depths between 20 and 40 µm. Below 40 µm, the ratio increases and then remains constant again. The decrease in the ratio indicates an increase in the number of [BO_4_] tetrahedral structural units relative to [BO_3_] triangular structural units. The trend in this ratio is consistent with the distribution of Cs concentration. It is well known that an increase in alkali metal content in borate glass favors the transformation of [BO_3_] structures into [BO_4_], and vice versa. Due to the migration of Cs ions from the surface to the interior under an electric field, the [BO_4_] structure on the glass surface transforms into [BO_3_]. Simultaneously, as the concentration of Cs ions increases internally, the [BO_3_] structure in the Cs‐enriched region of the glass (at depths ranging from 20 to 40 µm below the glass surface) transforms into [BO_4_].

**FIGURE 2:**
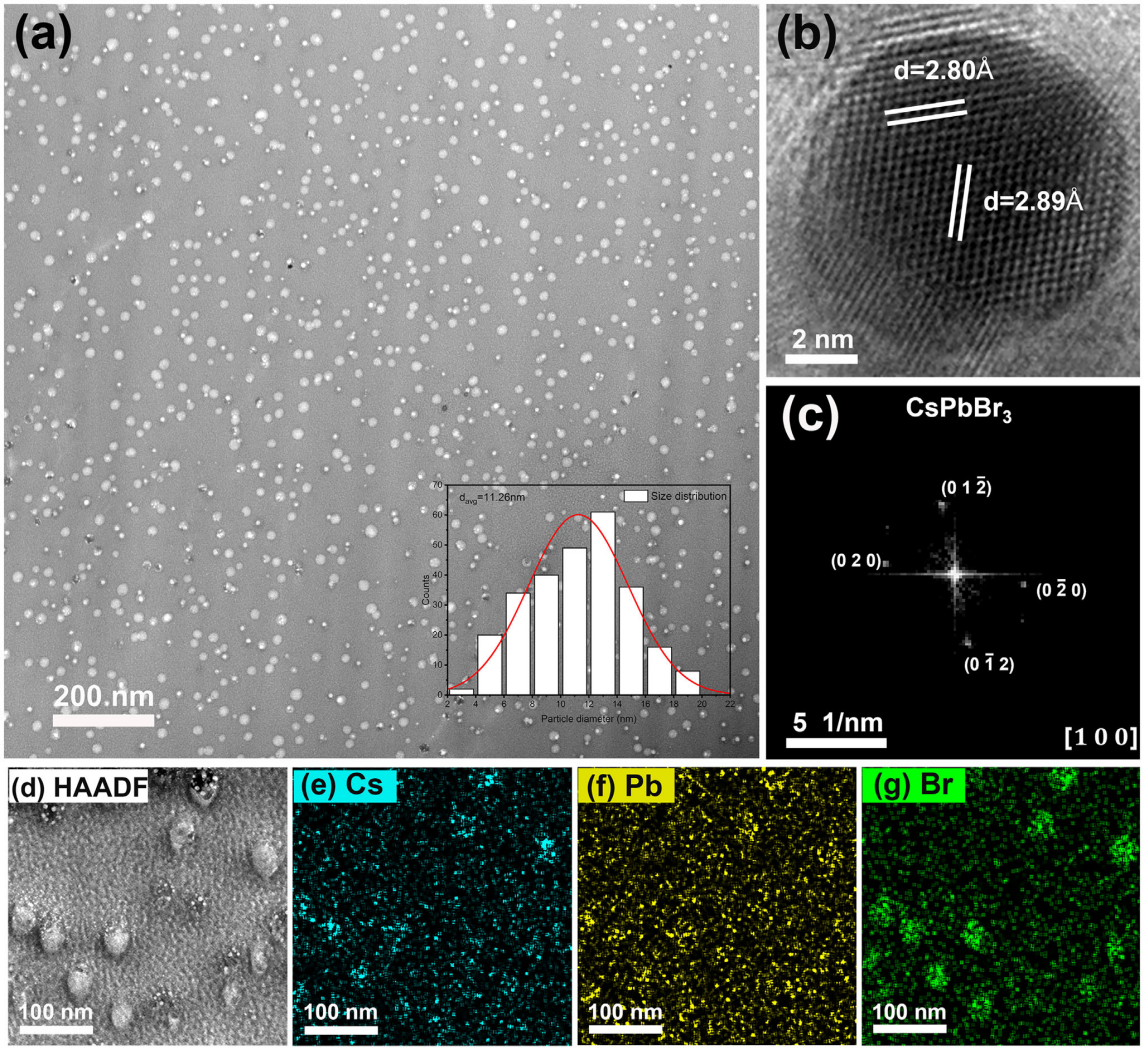
(a) High‐angle annular dark field (HAADF) image of the Cs ion‐enriched region in the glass after thermoelectric coupling treatment at 450°C and 900 V/mm for 1 h. The inset shows the size distribution of the nanocrystals. (b) High‐resolution transmission electron microscopy image of the nanocrystals and (c) the corresponding Fast Fourier transformation (FFT) pattern. (d) HAADF image of the glass and the corresponding elemental distributions of (e) Cs, (f) Pb, and (g) Br.

Figure 2a presents a high‐angle annular dark field (HAADF) image of the luminescent region of the glass obtained using high‐resolution transmission electron microscopy (HR‐TEM). The nanocrystals are uniformly distributed, with an average diameter of 11.26 nm. HR‐TEM analysis of the grains reveals lattice constants of 2.80 and 2.89 Å (Figure 2b), corresponding to the (020) and $\left(01\bar{2}\right)$ crystal planes of CsPbBr_3_ crystals, respectively. The corresponding fast Fourier transform (FFT) pattern aligns with the diffraction pattern along the [100] zone axis (Figure 2c). Elemental analysis indicates that the nanocrystal region is enriched in Cs, Pb, and Br elements (Figure 2d–g), further confirming the crystallization of CsPbBr_3_ PNCs in the glass under the influence of a thermoelectric field.

**FIGURE 3:**
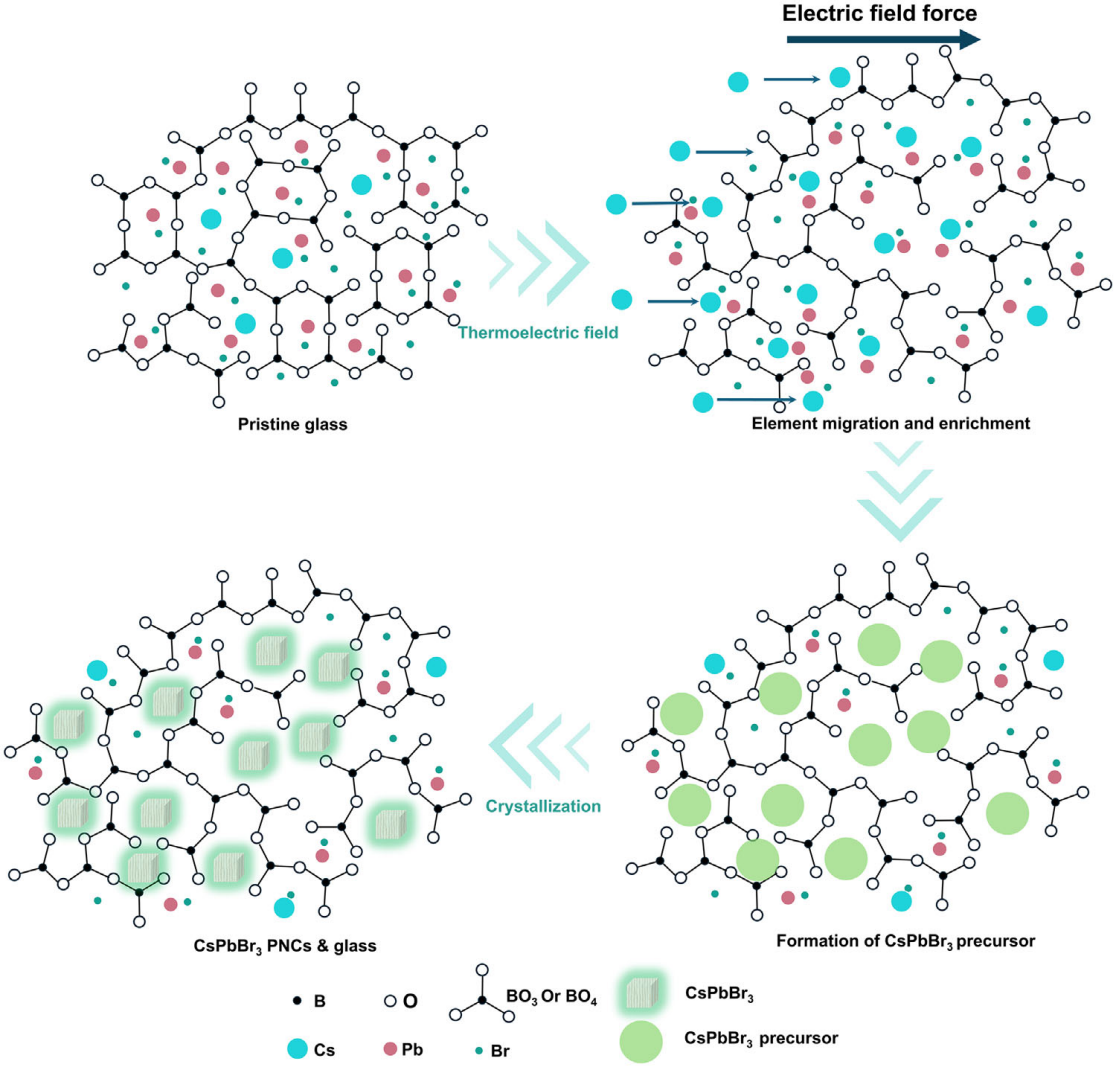
Schematic diagram of Cs^+^ ion migration during the thermoelectric treatment process and the crystallization process of CsPbBr_3_ PNCs in the glass network.

Based on the above discussion, the crystallization process of CsPbBr_3_ PNCs in glass under the influence of thermoelectric field is illustrated in Figure 3. Under the influence of the anode‐side electric field, Cs ions on the glass surface migrate towards the interior of the glass network, driven by their low binding energy with oxygen and low diffusion barrier. Conversely, divalent cations such as Pb^2+^ and Ca^2+^ have high binding energy with oxygen and correspondingly higher diffusion barriers in the glass network, making them difficult to migrate within the glass network under this electric field strength. The enrichment of Cs in glass leads to the transformation of a portion of [BO_3_] into [BO_4_]. The reconfiguration of the glass network structure leads to a more loosely packed arrangement, and the mild heat generated during the process of ion migration also facilitates the loosening of the glass network, thus establishing a favorable external environment for ion diffusion and the subsequent formation of CsPbBr_3_ PNCs. Furthermore, when Cs is enriched and reaches a certain concentration, it combines with Pb^2+^ and Br^−^ ions in the glass network to form nanoscale CsPbBr_3_ precursors. These precursors are subsequently converted into CsPbBr_3_ PNCs under certain conditions.

**FIGURE 4:**
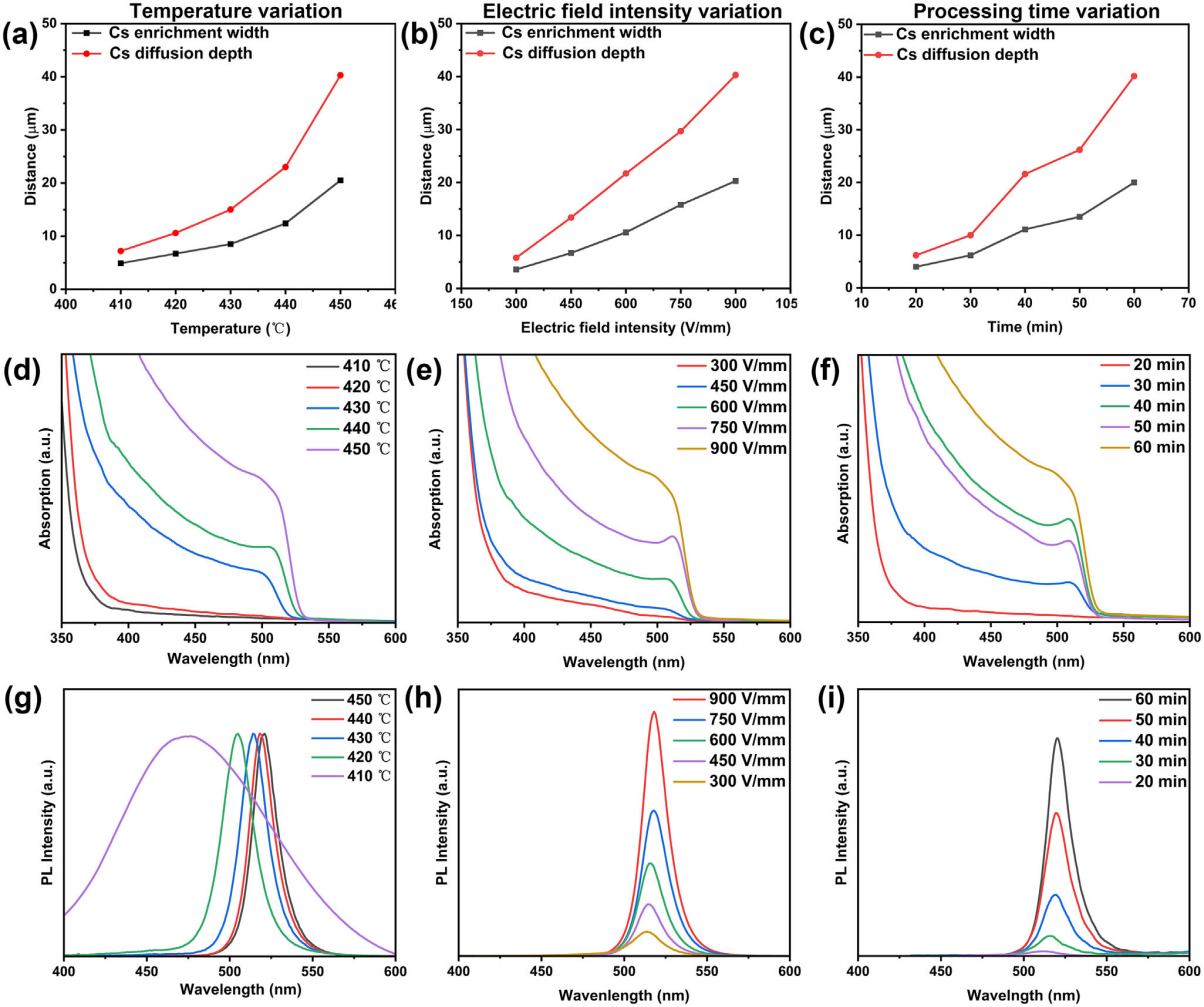
The depth and width of the cesium ion enrichment region after thermoelectric treatment under different (a) temperatures, (b) electric field strengths, and (c) durations. Absorption spectra of glass samples after thermoelectric treatment under different (d) temperatures, (e) electric field strengths, and (f) durations. Photoluminescence spectra of glass samples after thermoelectric treatment under different (g) temperatures, (h) electric field strengths, and (i) durations.

The thermoelectric field conditions (temperature, electric field strength, and durations) are crucial for ion migration and the crystallization of CsPbBr_3_ PNCs in glass. Figure 4a–c present statistical data on the depth and width of Cs enrichment in glass under various thermoelectric field conditions, including temperature, electric field strength, and durations (detailed ion distribution profiles with depth in glass are illustrated in Figure S7). The absorption and PL spectra of the thermoelectric treatment area in glass are depicted in Figure 4d–i, respectively. As the temperature rises, the depth and width of the Cs enrichment region expand nonlinearly (Figure 4a and Figure S8). According to the Arrhenius equation [20], the diffusion coefficient steadily rises with increasing temperature. Along with the enrichment of Cs under the thermoelectric field, the width of the enrichment area of Na and Br in the surface layer of glass also increases. Notably, at temperatures <440°C, the oxygen element remains essentially unchanged at different depths. However, when the temperature up to 450°C, oxygen elements exhibit significant enrichment in the glass surface, indicating that as temperature increases, oxygen gains sufficient energy to overcome the diffusion barrier and migrate. As the temperature continues to rise, the glass network undergoes dielectric breakdown in a short period of time under the influence of high voltage. At this juncture, the charge carriers acquire adequate energy from the electric field, leading to the breakdown of the glass interposed between the electrodes and the formation of a molten cavity, consequently causing a sharp decline in the voltage across the electrodes [35, 36]. The absorption properties of the thermoelectric treatment area are illustrated in Figure 4d. As the temperature rises, the absorption cutoff edge of the thermoelectric treatment area gradually redshifts, and the absorption coefficient continuously increases, indicating an increase in the size and quantity of CsPbBr_3_ PNCs. The PL spectra of the thermoelectric treatment area is depicted in Figure 4g. After thermoelectric treatment at 410°C, the PL peak of the treated area is at ∼475 nm, originating from the 6s6p‐6s^2^ transition of Pb^2+^ in the glass [28, 29], indicating that no CsPbBr_3_ PNCs have precipitated. Upon elevating the processing temperature to 420°C, the luminescence peak of the treated area becomes a narrow peak (∼505 nm), signifying the precipitation of CsPbBr_3_ PNCs in glass. As the processing temperature increases, the luminescence peak gradually shifts toward longer wavelengths to ∼520 nm, indicating that the size of CsPbBr_3_ PNCs gradually increases. Correspondingly, the color of the luminescent layer gradually changes from blue‐green to green (see Figure S9). These suggest that temperature serves as a pivotal energy source for the formation of CsPbBr_3_ PNCs, substantially influencing their formation and growth processes. Moreover, in comparison with the results presented in Figure S2, the crystallization temperature (420°C) or crystallization energy barrier of CsPbBr_3_ PNCs experiences a substantial reduction following the application of an electric field. This phenomenon can be ascribed, on one hand, to the enrichment of Cs ions, which elevates the likelihood of CsPbBr_3_ PNCs formation. On the other hand, it is also attributable to ion migration and the reconfiguration of the glass network structure, rendering the glass network more loosely structured and consequently facilitating the formation of CsPbBr_3_ PNCs.

Similarly, the electric field also affects the ions migration and the precipitation of CsPbBr_3_ PNCs in thermoelectrically treated glass. Figure 4b illustrates the distribution profile of ions in glass thermoelectrically treated under various voltage electric fields (300‐900 V/mm, 450°C, 60 min). Analogous to the effect of increasing temperature, as the electric field strength elevated, the diffusion depth of Cs and the width of its enriched region gradually increase. During the thermoelectric treatment, the electric field imparts additional energy for ion diffusion. At lower electric strengths, the energy acquired by Cs ions is insufficient to overcome the diffusion barrier, leading to a reduced Cs diffusion coefficient. However, as electric field strength escalates, Cs ions acquire ample energy to surmount the barrier, thereby augmenting the diffusion rate of Cs ions. Furthermore, the ions migration coefficients driven by an electric field were estimated by employ Nernst‐Einstein equation [34]:

$$\mu =\frac{D\cdot e}{H\cdot k_{B}\cdot T}$$

where *µ* is the the ion mobility, *D* is the ionic diffusion coefficient, *k_B_* is the Boltzmann constant, *T* is the temperature, *e* is the elementary charge, and *H* is the Haven ratio around 0.3 [34, 37]. Meanwhile, considering *v* =  *µ* *E*, and *x*  =  *vt*, ionic diffusion coefficient(*D*) can be written as:

$$D=\frac{v\cdot H\cdot k_{B}\;\cdot T}{E\cdot e}$$

where *E* is electric field intensity, *x* is ion migration distance, *v* is ion migration speed, and *t* is ion migration time. Based on the experimentally measured data, the ion diffusion coefficients of Cs under different external electric field intensities were calculated, and the results are listed in Table S1. The DC electric field provides an additional driving force for the directional ion migration, thereby effectively reducing the apparent activation energy. Strand et al. [38, 39] proposed a kinetic model to describe ion migration in glass under an electric field based on the Arrhenius relationship, expressed as:

$$D=D_{0}\exp\left(-\frac{E_{a}-\beta E}{k_{B}T}\right)\;\mathrm{or}\;\ln D=\frac{\beta E}{k_{B}T}+\left(\ln D_{0}-\frac{E_{a}}{k_{B}T}\right)$$

The Activation energy required for ion migration under the thermoelectric coupled field can be written as *E_a_*‐*β* *E*, where *β* is understood as the “field acceleration parameter” under the applied electric field intensity of *E*. The data presented in Table S1 exhibit a good fit when applied to the aforementioned formula, with the results illustrated in Figure S10. Therefore, it can be concluded that the ion mobility in this work exhibits an exponential relationship with the electric field.

The absorption and PL spectra of the thermoelectrically treated area are exhibited in Figure 4e,h, respectively. The absorption and PL peaks positions of samples treated with varying electric field strengths are essentially identical, suggesting that electric field strength exerts minimal influence on the growth of CsPbBr_3_ PNCs. The absorption and fluorescence intensities of CsPbBr_3_ PNCs escalate markedly with increasing electric field strength. This observation suggests that the extent of crystallization within the electric field region experiences a nonlinear and rapid augmentation as the electric field strength intensifies. The notable elevation in the content of CsPbBr_3_ PNCs in response to the electric field strength underscores that the electric field fosters the formation of CsPbBr_3_ PNCs, rather than merely facilitating ion migration. The findings presented in Figure 1f,g illustrate that the electric field exerts a regulatory influence on the glass network structure (specifically, the transformations between [BO_3_] and [BO_4_]). Moreover, previous research has indicated that the application of an electric field to glass promotes the generation of broken bonds within the glass network [40, 41], thereby rendering it more loosely structured. Consequently, as the electric field strength increases, accompanied by the breaking and subsequent reconfiguration of bonds within the glass network, the mobility of Cs, Pb, and Br ions within micro‐regions enhances, thereby facilitating the formation of CsPbBr_3_ PNCs more effectively. Also, as evident from the cross‐sectional photograph of the sample (Figure S9), the luminescent layer containing CsPbBr_3_ PNCs gradually broadens and deepens, while the luminescent color remains unchanged. It is noteworthy that with the escalation of electric field strength, the photoluminescence quantum yield (PL QY) of CsPbBr_3_ PNCs demonstrates a trend of initial increase (up to ∼94%) followed by a decrease (refer to Figure S11). This elevation in PL QY signifies a diminution of defects within CsPbBr_3_ PNCs, implying that the electric field is beneficial to some extent in improving the crystal structure of CsPbBr_3_ PNCs. The aforementioned regulatory impacts of temperature and electric field on CsPbBr_3_ PNCs indicate that the formation of CsPbBr_3_ PNCs within glass necessitates the synergistic interplay between thermal and electric fields. Only under the specific synergistic conditions of these two fields can CsPbBr_3_ PNCs precipitate in the region influenced by the electric field.

Furthermore, we analyzed the ion migration and crystallization behavior of CsPbBr_3_ PNCs in thermoelectrically treated glass at various durations. Under constant temperature and electric field conditions (450°C, 900 V/mm), the diffusion rate of ions in the glass remained essentially consistent. With the increase of durations, both the depth and width of the Cs‐enriched region increase linearly (see Figure 4c). Correspondingly, the absorption and PL peaks of the thermoelectrically affected region gradually intensified (see Figure 4f,i), indicating an increase in the number of CsPbBr_3_ PNCs. With the increase in depth and width of the Cs‐enriched region, the depth and width of the luminescent region also gradually increased. This phenomenon can be confirmed by the micro‐area PL spectra and the photographs of the glass cross‐section under ultraviolet excitation (Figures S9 and S12). It is worth noting that as the treatment duration increases, the corresponding luminescent wavelength shifts toward longer wavelengths, primarily due to the increase in nanocrystal size, indicating that the growth of CsPbBr_3_ PNCs is time‐dependent.

**FIGURE 5:**
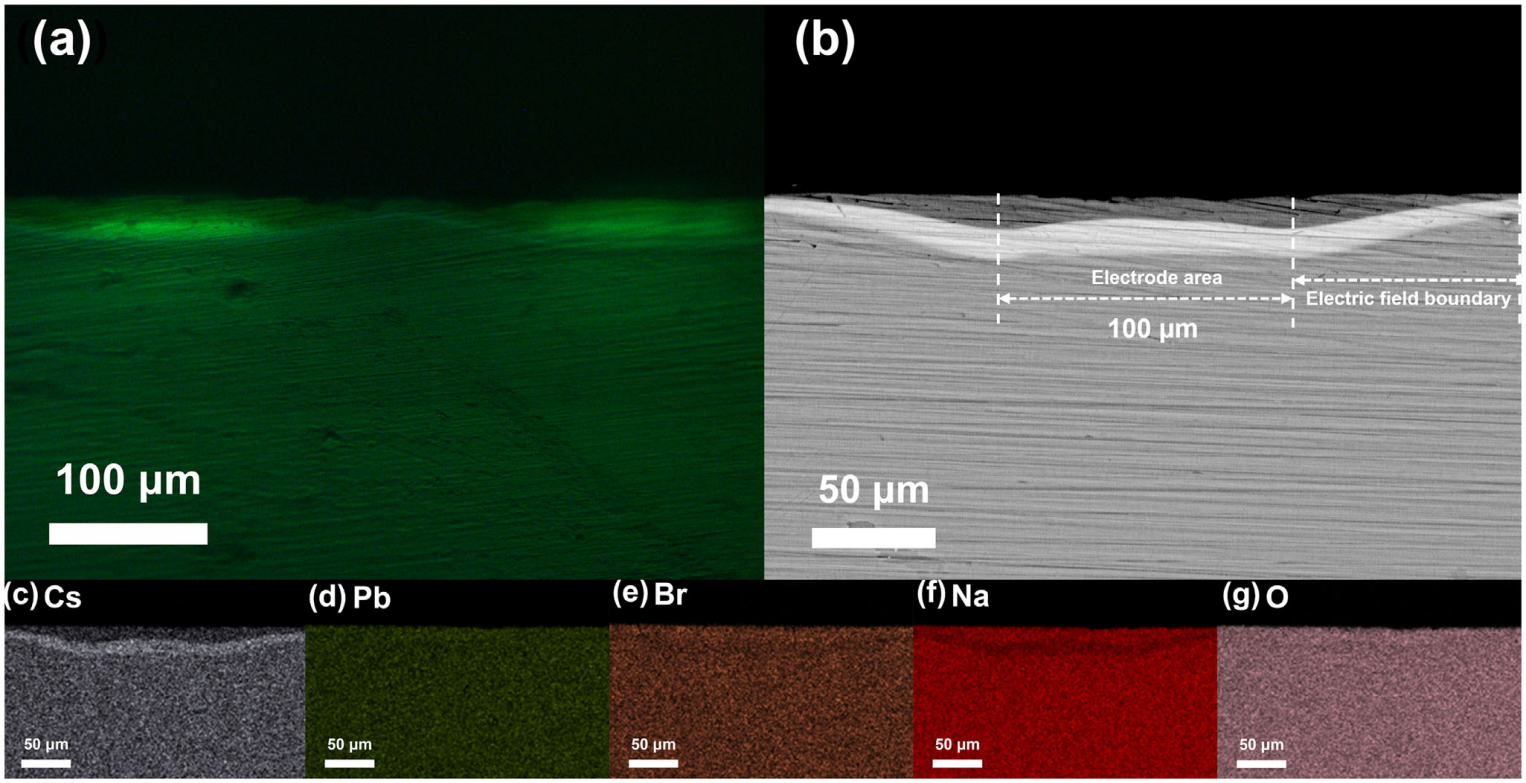
(a) Micrograph of the cross‐section of the glass with the luminescent dot matrix pattern after thermoelectric treatment (450°C, 900 V/mm, 40 min). (b) SEM backscatter image of the cross‐section of the dot matrix region in the glass, as well as the elemental distribution of (c) Cs, (d) Pb, (e) Br, (f) Na, and (g) O in this region.

To achieve patterned distribution of CsPbBr_3_ PNCs in glass, we utilized patterned electrodes to perform thermoelectric treatment on the glass sample. Figure 5a depicts the cross‐section of the glass area under ultraviolet excitation after thermoelectric treatment (450°C, 900 V/mm, 40 min) with the dot matrix (dot size: 100 µm) patterned electrodes, revealing luminescent regions with a width of ∼135 µm and a thickness of ∼20 µm, spaced apart. Figure 5b presents a SEM backscattered image of the cross‐section of the dot region. The bright areas in the image originate from the enrichment of elements with high atomic number under the influence of local electric fields. The elemental analysis results in Figure 5c reveal that this area is predominantly enriched in Cs ions, while Na, Br, and O are relatively scarce, aligning with the aforementioned elemental distribution results. Notably, the width of the bright area significantly exceeds the contact area between the electrode and the glass. This suggests that the edge of the anode electrode also influences the migration and enrichment of Cs ions in the glass. Furthermore, the enrichment thickness diminishes with increasing distance from the electrode edge, conforming to the distribution law of charge potential.

**FIGURE 6:**
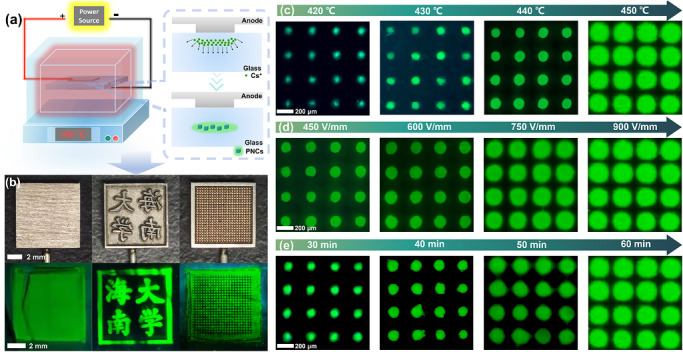
(a) Schematic diagram of inducing CsPbBr_3_ nanocrystal distribution through thermoelectric coupling treatment. (b) Different electrode structures and their corresponding luminescent patterns (squares, Chinese characters, and dot matrices). Dot matrix luminescent patterns in glass after thermoelectric treatment at different (c) temperatures, (d) electric field strengths, and (e) durations.

In the glass area in contact with the patterned electrode, Cs ions migrate and accumulate, inducing the precipitation of CsPbBr_3_ PNCs (as shown in Figure 6a). Figure 6b displays the electrode with a patterned structure, and the corresponding photos of the glass upon thermoelectric treatment (450°C, 900 V/mm, 60 min) using these electrodes under irradiation of ultraviolet light. The pattern of square, Chinese characters, and dot matrix in the glass luminescent area in the figure perfectly matches the pattern of the electrodes (as shown in Figure 6b). Furthermore, we explored the thermoelectric treatment of glass using electrodes with micro dot matrix patterns (dot size:100 µm). Figure 6c–e depict the variations in the dot matrix patterns in the glass under different thermoelectric treatment conditions, specifically temperature, electric field strength, and treatment duration. Evidently, as temperature, electric field strength, and duration increase, the size of the dots in the matrix gradually enlarges, which can be effectively controlled within the range of ∼50‐240 µm.

The edge effect of the anode electrode plays a crucial role in determining the size of dot area in the glass. At low temperatures, weak electric field, and short durations, the enrichment thickness of Cs in the glass is narrow. Consequently, the element enrichment induced by the edge electric field is weaker or even negligible. As temperature, electric field strength, and treatment time increase, the degree of element enrichment caused by the edge electric field gradually intensifies, thereby inducing the precipitation of CsPbBr_3_ PNCs and enlarging the size of the dot luminescent regions (Figure 6c–e). To quantitatively clarify the fidelity between the dot pattern on the electrode and the luminescent pattern within the glass, a statistical analysis was conducted on the sizes of luminescent dots in the glass under various thermoelectric treatment conditions, as depicted in Figure S13 and Tables S2–S4. Notably, under the thermoelectric treatment conditions of (430°C, 900 V/mm, 60 min) and (450°C, 900 V/mm, 30 min), a high degree of consistency was observed between the sizes of the luminescent dots and those of the dots on the electrode. Furthermore, under identical thermoelectric treatment conditions, the dot diameter, spacing, and luminous intensity of the dot matrix patterns across different treatment batches exhibited excellent uniformity, consistency, and reproducibility (Figure S14). In comparison with other patterning techniques (Table S5), the thermoelectric synergistic method enables the controllable distribution of CsPbBr_3_ PNCs within a stable glass matrix, along with excellent optical properties, through a straightforward single‐step process. These findings demonstrate that thermoelectric treatment is an effective method for achieving patterned distribution of CsPbBr_3_ PNCs in glass, presenting broad application prospects in displays and other optical applications.

## 3 Conclusion

In summary, the directional migration and enrichment of Cs^+^, Na^+^, Br^−^, and O^2−^ ions in glass were achieved through thermoelectric coupling treatment, thereby inducing the precipitation of CsPbBr_3_ PNCs in a localized area. The migration of Cs induces a transition between [BO_3_] and [BO_4_] in the glass network. The enrichment of Cs under the influence of an electric field is the primary reason for the precipitation of CsPbBr_3_ PNCs. These ions‐enriched regions are exhibited on the micrometer scale, and their depth and width are dependent on temperature, electric field strength, and time. Enhancing these factors contributes to increase the depth and width of the Cs enrichment region. By adjusting the temperature, electric field strength, and duration of thermoelectric treatment, the width and depth of the luminescent region comprise of CsPbBr_3_ PNCs can be precisely controlled at the micrometer scale. Furthermore, patterned distribution of CsPbBr_3_ PNCs in glass can be achieved using patterned electrodes and thermoelectric treatment. With electrodes featuring a micro‐dot matrix pattern (dot size: 100 µm), the dot size in the matrix can be effectively controlled between approximately 50–240 µm by adjusting the thermoelectric treatment conditions. This method provides a new approach for precise printing of CsPbX_3_ PNCs in glass samples and demonstrates broad application prospects in optical fields such as Micro‐LEDs, information storage, and biological imaging.

## 4 Experimental

In this study, the original glass was prepared using the traditional melting and quenching method, with a composition of 50B_2_O_3_‐27ZnO‐4Al_2_O_3_‐2CaO‐4Cs_2_O‐5PbBr_2_‐14NaBr (in mol %). The weighed raw materials were finely ground in a mortar according to the stoichiometric ratio, and heated in a high‐temperature furnace at 1200°C for 15 min. After melting, the molten glass was poured into a brass mold for quenching. Subsequently, the obtained glass was transferred to a muffle furnace and annealed at 400°C for 2 h to release the thermal stress generated during quenching. The annealed glass was cut and polished into glass pieces with dimensions of 1.5 × 1.5 × 0.2 cm^3^ for the subsequent thermoelectric treatment.

Thermoelectric coupling treatment is conducted in a specially designed device. Specifically, the device comprises a high‐voltage direct current (DC) power supply, a muffle furnace, and two high‐temperature‐resistant electrodes (Size: 8 mm × 8 mm × 2 mm) made of nickel‐chromium (80Ni20Cr) alloy (see Figure 1a). All thermoelectric coupling experiments were carried out under a continuous constant‐voltage direct current (DC) mode. A high‐voltage transformer (TDGC2‐5KVA) was employed to boost the 220 V voltage to the desired high voltage level, which was subsequently channeled through a high‐voltage silicon stack bridge rectifier circuit, a filter capacitor, and a voltage regulator (7DG‐5K) to achieve a stable DC output. During the thermoelectric coupling treatment process, the glass sample is placed between the cathode and anode electrodes, and the sample is heated in the muffle furnace at a rate of 10°C/min. To guarantee optimal contact between the electrode and the glass sample, two customized high‐purity alumina ceramic plates (2 mm thick) are employed to secure the electrode plate onto both the upper and lower surfaces of the glass, thereby constraining its movement within the glass plane. Furthermore, an 80 × 80 × 5 mm^3^ alumina plate is utilized to restrict the vertical displacement of the electrode plate, ensuring its horizontal alignment, as depicted in Figure S3. The furnace thermocouple is closely affixed to the high‐thermal‐conductivity alumina plate, ensuring that the temperature of the glass sample accurately reflects the preset temperature of the electric furnace. The patterned electrode sheet is produced by laser etching an 8 × 8 × 2mm^3^ electrode sheet to create customized raised patterns. For the dot matrix pattern electrode, the size of the dot pattern is 100 µm, the spacing between dots is 300 µm, the depth of the pattern raised is 300 µm, and the processing accuracy is ±5 µm. Due to the good high‐temperature resistance and corrosion resistance of nickel‐chromium alloy, the surface of the patterned electrode underwent no special treatment. Once reaching the set temperature (410‐450°C), the power supply is turned on and different intensities of direct current (DC) electric fields (300‐900 V/mm, corresponding to 600–1800 V) are applied, respectively, until the thermal field treatment time (20‐60 min) is over.

The phase state of the glass sample was analyzed using an X‐ray diffractometer (XRD, Rigaku Smart Lab 9 kW, Japan) equipped with Cu Kα radiation (wavelength = 1.54 Å) and a scanning rate of 2° per minute. The structure of the sample was characterized using a confocal Raman microscope (Renishaw inVia Qontor, UK) with a laser wavelength of 633 nm. Additionally, the micro‐area fluorescence of the thermoelectric field treated glass was tested using the fluorescence mode of the confocal Raman microscope (Renishaw inVia Qontor, UK) with a laser wavelength of 405 nm. The morphology and elemental distribution of the glass cross‐section were analyzed using a scanning electron microscope (SEM, Thermoscientific Quattro S, USA) equipped with an energy dispersive X‐ray spectrometer (EDS, Elect Super, EDAX, USA). The morphology and elemental distribution of nanocrystals in the glass were analyzed using a high‐resolution transmission electron microscope (HR‐TEM, Talos F200X G2, USA) operating at 200 kV and equipped with an energy dispersive X‐ray spectrometer. The absorption spectra of the glass samples were recorded using a UV/VIS/NIR spectrophotometer (Lambda 750s, PerkinElmer, USA), and the photoluminescence (PL) spectra were collected using a spectrometer (FLS1000, France) under excitation by 365 nm light.

## Author Contributions

**Zhiheng Miao**: data curation, investigation, methodology, validation, writing – original draft. **Yao Zhou**: conceptualization, methodology, supervision, writing – original draft, writing – review and editing. **Minzhi Li**: data curation, investigation. **Zhe Wang**: formal analysis, software. **Xiaolong Yu**: formal analysis, resources. **Jianlin Li**: resources, supervision, visualization. **Changjiu Li**: conceptualization, funding acquisition, project administration, resources, writing – review and editing

## Funding

National key R&D program of China (2022YFB3705703).

## Conflicts of Interest

The authors declare no conflicts of interest.

## Supporting information




**Supporting File**: advs75827‐sup‐0001‐SuppMat.docx.

## Data Availability

The data that support the findings of this study are available from the corresponding author upon reasonable request.
