# Supplementary material for: Precisely Printing Perovskite Nanocrystals in Glass via Thermoelectric Synergistic Effect
Source: Adv Sci (Weinh). 2026 May 26;13(46):e75827. doi: 10.1002/advs.75827 (PMC13335932; doi:10.1002/advs.75827)
Supplement: Supplementary file 1 [file ADVS-13-e75827-s001.docx]

Supplementary Materials

**Precisely printing perovskite nanocrystals in glass via thermoelectric synergistic effect**

*Zhiheng Miao^#,1,2^, Yao Zhou^#*,1,2,3^, Minzhi Li^1,2^, Zhe Wang^1,2^, Xiaolong Yu^1,2^, Jianlin Li^1,2^, Changjiu Li^*1,2^*

^1^ State Key Laboratory of Tropic Ocean Engineering Materials and Materials Evaluation, Hainan University, Haikou 570228, China

^2^ College of Materials Science and Engineering, Hainan University, Haikou 570228, China

^3^ School of Ecology, Hainan University, Haikou 570228, China

^*^ Corresponding authors E-mail: Yao_Zhou@hainanu.edu.cn (Y. Zhou); lichangjiu@hainanu.edu.cn (C. Li)

^#^ These authors contributed to the work equally and should be regarded as co-first authors.

**Figure S1**. XRD patterns of glass samples after heat-treated at 470 ℃ for 0.5, 1, and 3 hours.

**Figure S2**. (a)Photos, (b) Absorption spectra, and (c) photoluminescence spectra of glass samples after heat treatment for 1 hour at various temperatures (440 ℃, 450 ℃, 460 ℃, and 470 ℃).

**Figure S3**. A schematic cross-sectional illustration of the experimental apparatus for glass thermoelectric treatment, depicting the detailed layout of the glass specimen, upper and lower electrodes, as well as the supporting fixtures.

**Figure S4** The temporal variation of current in the circuit, under the thermoelectric treatment conditions of 450 ℃ and 900 V/mm

**Figure S5**. Scanning electron microscopy (SEM) backscattered images of glass cross-sections obtained under thermoelectric treatment conditions, specifically with an electric field strength of 900 V/mm, a temperature of 450 ℃, and a duration of 1 hour. Based on Z-contrast, bright areas indicate Cs-enriched regions, whereas dark areas signify regions with a relative scarcity of Cs.

**Figure S6**. Raman spectra of thermoelectrically treated samples at various depths (i.e. 14, 28, and 42 μm), along with their fitted peaks corresponding to BO_4_ stretching vibration and BO_3_ stretching vibration, respectively.

**Figure S7**. The relative concentration of elements in the glass with depth after thermoelectric treatment under different (a) temperatures, (b) electric field strengths, and (c) durations. For thermoelectric treatment at various temperatures, the electric field and duration are maintained at 900 V/mm and 60 minutes, respectively; Similarly, for thermoelectric treatment under varying electric field intensities, the temperature and duration are maintained at 450 ℃ and 60 minutes, respectively; For thermoelectric treatments conducted for different durations, the temperature and electric field strength were maintained constant at 450 ℃ and 900 V/mm, respectively.

**Figure S8**. SEM backscatter images of glass cross-sections after thermoelectric treatment (900 V/mm, 60min) at 410, 420, 430, 440, and 450 ℃, respectively. Based on Z-contrast, the bright areas indicate Cs-enriched regions.

**Figure S9**. Cross-sectional images of glass samples after thermoelectric treatment under varying conditions of (a) temperature, (b) electric field strength, and (c) treatment duration. All photos of the samples were taken under exposure to 365 nm ultraviolet light.

| **Table S1. Variations in the diffusion coefficient of Cs^+^ ions under different electric field.** | | | | | |
| --- | --- | --- | --- | --- | --- |
| Electric field intensity (V/mm) | 300 | 450 | 600 | 750 | 900 |
| Diffusion coefficient of Cs^+^ ions (m^2^/s) | 9.5×10^-17^ | 1.2×10^-16^ | 1.6×10^-16^ | 1.7×10^-16^ | 2.0×10^-16^ |

**Figure S10.** The fitting plot of the natural logarithm of Cs^+^ ionic diffusion coefficient (ln*D*) versus electric field intensity (*E*), with fitting degree R^2^=0.95456.

**Figure S11**. The Photoluminescence Quantum Efficiency (PL QY) of the sample following treatment at 450 ℃ under varying electric field intensities. (a-e) illustrate the PL QY of the sample subsequent to treatment with electric field intensities of 300, 450, 600, 750, and 900 V/mm, respectively; (f) depicts the variation in the sample's PL QY as a function of electric field intensity.

**Figure S12**. Photoluminescence spectra at different depths in glass after thermoelectric treatment (450 ℃, 900 V/mm) for (a) 30 min, (b) 40 min, (c) 50 min, and (d) 60min.

**Table S2. Dot diameters under different temperatures.**

| Treatment temperature (℃) | 420 | 430 | 440 | 450 |
| --- | --- | --- | --- | --- |
| Dot diameter (μm) | 54.5±9.9 | 101.2±7.0 | 125.1±7.4 | 236.9±7.7 |

**Table S3. Dot diameters under different electric field intensities.**

| Electric field (V/mm) | 450 | 600 | 750 | 900 |
| --- | --- | --- | --- | --- |
| Dot diameter (μm) | 122.3±7.0 | 149.3±6.8 | 198.9±7.5 | 236.9±7.7 |

**Table S4. Dot diameters under different time.**

| Treatment duration (min) | 30 | 40 | 50 | 60 |
| --- | --- | --- | --- | --- |
| Dot diameter (μm) | 97.0±7.5 | 135.9±8.0 | 190.9±8.5 | 236.9±7.7 |

**Figure S****13**. Size statistics of dot matrix patterns realized via electrode-assisted thermoelectric treatment: Dependence of dot size variation on (a) temperature (420 - 450 ℃), (b) processing duration (30 - 60 minutes), and (c) electric field strength (450 - 900 V/mm).

**Figure S****14.** Statistical data regarding dot diameter, spacing, and photoluminescence intensity across five independent batches. The consistent values confirm the high reproducibility of the patterning method.

**Table S5. Comprehensive Performance Comparison of Various PQD Patterning Methods**

| **Method** | **Processing steps** | **Resolution** | **Processing efficiency** | **PL performance** | **Reference** |
| --- | --- | --- | --- | --- | --- |
| Photolithography | Coating of NCs and bisazide additives→UV exposure with photomask→Developing, Repeat for aditional patterning | Determined by photomaks, ptimal dot size: 2–100 μm. | Multiple steps and high time-cost | Prolonged UV irradiation reduces PLQY of pre-embedded PNCs to 60% of initial value; subsequent ligand exchange restores it to 76%. | [1,2] |
| Direct Photolithography | Perovskite precursor coating → Masked UV exposure→Developing→Annealing & in situ PQDs patterning | Determined by photomask used, 20 μm-40 μm pixel size demonstrated | Multiple steps and high time-cost | PLQY: Blue 17 %; Green 87 %; Red 58 % | [3] |
|  | Precursor-polymer coating→Masked UVC exposure→In-situ PQDs growth | Determined by photomaks, Ptimal dot size: 20–100 μm | Direct photolithography, development/etching-free, relatively high processing efficiency | PLQY~51% | [4,5] |
| Inkjet printing | Preparation of ink contianing NCs→Printing | Resolution limited bythe size of nozzle and the rheological properties of the ink, optimal dot size: 20–40 μm. Coffee-ring effect persists in large-sized dot fabrication. | Printing speed onthe order of μm /s | PLQY up to 92%, with potential ink degradation during printing | [6,7] |
| Femtosecond laser induced crystallization | Glass containing nanocrystals precursor→fs-laser irradiation | The feature size (10–50 μm) is determined by the femtosecond laser power and irradiation time. | Processing time is related to the number of irradiation dots. Each dot lasts for 400 to 1000 milliseconds. | Low PLQY ~10-20%, reduced optical quality of glass matrix due to laser induced thermal Effect; excessive laser irradiation causes residual stress & impairs glass optical performance | [8-11] |
| Laser ablation | Spin-coating→Film formation→Laser writing | Etching dimension defined by laser wavelength; linewidth down to 200 nm via optimized power and scanning speed. Etching resist may degrade resolution. | 10-30 mm/s write | Dependent on the polymer andprecusors, high PLQY demonstrated inthe work | [12,13] |
| **Electro-thermal field- induced crystallization** | **Glass containing nanocrystals precursor→Thermoelectric synergistic** | **Determined by the pattern size on the electrode and the thermoelectric processing conditions, the pixel size range in this work is from 50 to 240 μm** | **One-step processing, with a duration of 20-60 min** | **PLQY up to~94%, influenced by the thermoelectric treatment conditions** | **This Work** |

**References**

[1]	D. Liu, K. Weng, S. Lu, F. Li, H. Abudukeremu, L. Zhang, Y. Yang, J. Hou, H. Qiu, Z. Fu, X. Luo, L. Duan, Y. Zhang, H. Zhang, J. Li, *Science Advances* **2022** *8* (11), eabm8433, <https://doi.org/10.1126/sciadv.abm8433>.

[2]	W. Sun, F. Li, J. Tao, P. Li, L. Zhu, J. Li, J. Lv, W. Wang, J. Liang, H. Zhong, *Nanoscale* **2022**, *14* (16), 5994, <https://doi.org/10.1039/D2NR01115H>.

[3]	P. Zhang, G. Yang, F. Li, J. Shi, H. Zhong, *Nature Communications* **2022**, 13 (1), 6713, <https://doi.org/10.1038/s41467-022-34453-9>.

[4]	W. Li, M. Wu, H. Chen, P. Zhang, Z. Cai, S. Cai, F. Li, *Small Struct.* **2024**, *5* (9), 2400078, <https://doi.org/10.1002/sstr.202400078>.

[5]	S.-Y. Liang, Y.-F. Liu, H.-J. Zhang, Z.-K. Ji, H. Xia, *ACS Applied Materials & Interfaces* **2022**, *14* (41), 46958, <https://doi.org/10.1021/acsami.2c11870>.

[6]	Z. Bao, J.-W. Luo, Y.-S. Wang, T.-C. Hu, S.-Y. Tsai, Y.-T. Tsai, H.-C. Wang, F.-H. Chen, Y.-C. Lee, T.-L. Tsai, R.-J. Chung, R.-S. Liu, *Chemical Engineering Journal* **2021**, *426*, 130849, [https://doi.org/10.1016/j.cej.2021.130849](https://doi.org/https://doi.org/10.1016/j.cej.2021.130849).

[7]	J. Cai, W. Lai, Y. Chen, X. Zhang, Y. Zheng, W. Zhang, X. Chen, Y. Ye, S. Xu, Q. Yan, T. Guo, E. Chen, *Laser & Photonics Reviews* **2024**, *18* (10), 2400298, [https://doi.org/10.1002/lpor.202400298](https://doi.org/https://doi.org/10.1002/lpor.202400298).

[8]	K. Sun, D. Tan, X. Fang, X. Xia, D. Lin, J. Song, Y. Lin, Z. Liu, M. Gu, Y. Yue, J. Qiu, *Science* **2022**, *375* (6578), 307, <https://doi.org/10.1126/science.abj2691>.

[9]	Y. Hu, Y. Ye, W. Zhang, K. Li, Y. Zhou, Y. Zhang, Z. Deng, J. Han, X. Zhao, C. Liu, *Journal of Materials Science & Technology* **2023**, *150*, 138, [https://doi.org/10.1016/j.jmst.2022.11.055](https://doi.org/https://doi.org/10.1016/j.jmst.2022.11.055).

[10]	X. Li, K. Sun, J. Wu, Y. Liu, L. Li, Z. Xiao, Z. Li, X. Liu, B. Xu, J. Qiu, D. Tan, *Laser & Photonics Reviews* **2024**, *18* (5), 2301244, [https://doi.org/10.1002/lpor.202301244](https://doi.org/https://doi.org/10.1002/lpor.202301244).

[11]	J. Wu, X. Li, K. Sun, K. Gao, C. Chen, J. Qiu, D. Tan, *Laser & Photonics Reviews* **2025**, *19* (10), 2401742, [https://doi.org/10.1002/lpor.202401742](https://doi.org/https://doi.org/10.1002/lpor.202401742).

[12]	W. Zhan, L. Meng, C. Shao, X.-g. Wu, K. Shi, H. Zhong, *ACS Photonics* **2021**, *8* (3), 765, <https://doi.org/10.1021/acsphotonics.1c00118>

[13]	S.-Y. Liang, Y.-F. Liu, H.-J. Zhang, Z.-K. Ji, H. Xia, *ACS Applied Materials & Interfaces* **2022**, *14* (41), 46958, <https://doi.org/10.1021/acsami.2c11870>.
